# Leptospirosis as a cause of fever associated with jaundice in the Democratic Republic of the Congo

**DOI:** 10.1371/journal.pntd.0009670

**Published:** 2021-08-17

**Authors:** Patrick Mukadi Kakoni, Yannick Munyeku Bazitama, Jean Raphael Nepomuceno, Elisabeth Pukuta-Simbu, Francois Kawhata Mawika, Gracia Kashitu Mujinga, Luigi Palla, Steve Ahuka-Mundeke, Jean-Jacques Muyembe Tamfum, Nobuo Koizumi, Yoshinao Kubo, Koya Ariyoshi, Chris Smith

**Affiliations:** 1 Department of Global Health, School of Tropical Medicine and Global Health, Nagasaki University, Nagasaki, Japan; 2 Institut National de Recherche Biomedicale (INRB), Kinshasa, the Democratic Republic of the Congo; 3 Faculté de Médecine, Université de Kinshasa, Kinshasa, the Democratic Republic of the Congo; 4 Program for Nurturing Global Leaders in Tropical and Emerging Communicable Diseases, Graduate School of Biomedical Sciences, Nagasaki University, Nagasaki, Japan; 5 Department of Clinical Tropical Medicine, Institute of Tropical Medicine, Nagasaki University Graduate School of Biomedical Science, Nagasaki, Japan; 6 Department of Bacteriology I, National Institute of Infectious Diseases (NIID), Tokyo, Japan; 7 Department of Public Health and Infectious Diseases, University of Rome La Sapienza, Roma, Italy; 8 Department of Clinical Research, London School of Hygiene and Tropical Medicine, London, United Kingdom; Universidade Federal de Pelotas, BRAZIL

## Abstract

**Background:**

Fever with jaundice is a common symptom of some infectious diseases. In public health surveillance within the Democratic Republic of the Congo (DRC), yellow fever is the only recognized cause of fever with jaundice. However, only 5% of the surveillance cases are positive for yellow fever and thus indicate the involvement of other pathogens. *Leptospira* spp. are the causative agents of leptospirosis, a widespread bacterial zoonosis, a known cause of fever with jaundice. This study aimed to determine the seropositivity of anti-*Leptospira* antibodies among suspected yellow fever cases and map the geographical distribution of possible leptospirosis in the DRC.

**Methods:**

We conducted a retrospective study using 1,300 samples from yellow fever surveillance in the DRC from January 2017 to December 2018. Serum samples were screened for the presence of IgM against *Leptospira* spp. by a whole cell-based IgM ELISA (Patoc-IgM ELISA) at the Institut National de Recherche Biomedicale in Kinshasa (INRB) according to World Health Organization (WHO) guidance. Exploratory univariable and multivariable logistic regression analyses were undertaken to assess associations between socio-demographic factors and the presence of *Leptospira* IgM.

**Results:**

Of the 1,300 serum samples screened, 88 (7%) showed evidence of IgM against *Leptospira* spp. Most positive cases (34%) were young adult males in the 20–29-year group. There were statistically significant associations between having *Leptospira* IgM antibodies, age, sex, and living area. Observed positive cases were mostly located in urban settings, and the majority lived in the province of Kinshasa. There was a statistically significant association between seasonality and IgM *Leptospira* spp. positivity amongst those living in Kinshasa, where most of the positive cases occurred during the rainy season.

**Conclusions:**

This study showed that leptospirosis is likely an overlooked cause of unexplained cases of fever with jaundice in the DRC and highlights the need to consider leptospirosis in the differential diagnosis of fever with jaundice, particularly in young adult males. Further studies are needed to identify animal reservoirs, associated risk factors, and the burden of human leptospirosis in the DRC.

## Author summary

Leptospirosis is an important bacterial zoonosis with a worldwide distribution. Each year there are an estimated one million cases, with about 60,000 deaths. The true burden of the disease, however, is unknown. The burden of leptospirosis is probably underestimated due to the lack of specific clinical symptoms and diagnostic techniques that are not readily available. Clinical diagnosis of leptospirosis is difficult because of the diversity of symptoms, ranging from asymptomatic forms to severe multivisceral icteric states. Differential diagnoses with infections presenting with fever or fever and jaundice are numerous and may mislead the clinician. Leptospirosis is considered endemic in sub-Saharan Africa and is known to cause fever with jaundice in African countries; however, for most countries, available epidemiologic data are scarce, including in the Democratic Republic of Congo (DRC). An improved understanding of the epidemiology of leptospirosis will improve clinical management, lead to policy formulation, and have implications for national surveillance of infectious diseases in these countries. We conducted a retrospective seroepidemiological study to extend the description of the pathogens responsible for fever with jaundice in the DRC and to clarify the circulation of possible leptospirosis in the country. This study showed that leptospirosis is a likely cause of fever with jaundice in the DRC.

## Introduction

Fever with jaundice is a frequent syndrome in infectious diseases. In sub-Saharan Africa, fever with jaundice can occur in the presence of parasitic (malaria, toxoplasmosis, schistosomiasis), bacterial (typhoid fever, leptospirosis, *Borrelia burgdorferi*, scrub typhus), or viral (viral hepatitis, Ebola virus, hantavirus, herpes virus) infections. Understanding the local epidemiology of these infections and public health problems in the region is helpful for the formulation of differential diagnoses[1–3].

Yellow fever remains a public health issue despite the availability of a safe and effective vaccine. Most endemic countries implement yellow fever surveillance due to its severity and the risk of widespread outbreaks[4]. In 2003 the Democratic Republic of the Congo (DRC) established a yellow fever surveillance system with support from the World Health Organization (WHO), in which health districts and health facilities are responsible for reporting suspected cases of yellow fever. Operationally, any case of fever with jaundice is considered a suspected case of yellow fever, and in these instances blood samples are collected and tested for yellow fever antibodies (immunoglobulin M, IgM) in a qualified laboratory.

However, in the DRC, around 5% of suspected cases of fever with jaundice reported at the Institut National de Recherche Biomédicale (INRB) in Kinshasa from 2003 to 2010 were positive for yellow fever, indicating the involvement of other pathogens as well as non-infectious causes. Studies conducted in the DRC by Makiala et al. to identify other causes of fever and jaundice among suspected yellow fever cases have shown the implications of viruses such as hepatitis B (26%), hepatitis C (2%), herpesvirus (26%), dengue, and chikungunya (8%) [5, 6]. Even so, these studies focused only on viral causes, while the differential diagnosis of fever with jaundice in this setting includes both bacterial and parasitic causes, thus requiring further evaluation.

Studies using serological and genomic methods in the Central African Republic, Sierra Leone, Ivory Coast, and Burkina Faso have implicated *Leptospira* as a cause of fever with jaundice in patients with suspected yellow fever [7–11]. Leptospirosis is an emerging zoonotic disease of global importance with a worldwide distribution and is caused by infection with pathogenic bacteria of the genus *Leptospira*. More than one million cases of leptospirosis occur worldwide each year, with almost 60,000 deaths [12]. Clinical pictures of leptospirosis vary from asymptomatic infection to a severe multi-visceral failure combining renal and hepatic disorders, termed Weil’s disease. Fever with conjunctival jaundice is described in manifestations of severe forms of leptospirosis [13]. African countries have favorable environmental characteristics for the spread of leptospirosis (rainfall, animal exposure, flooding, rapid urbanization). However, the incidence and prevalence of the disease are not well known.

Data on leptospirosis epidemiology from African countries are limited, and the available data are from North and West Africa. Observations suggest that leptospirosis may be much more widespread in this region of the world than previously thought [14–16]. Data regarding the Central Africa region are scarce, and little is known about the prevalence and burden of leptospirosis in the DRC. Publications report human and dog cases during the colonial period plus a recent accidental discovery of the involvement of leptospirosis in an epidemic of pneumonic plague [17, 18]. Data are needed to estimate the burden of the disease and for the implementation of prevention and control strategies.

To fill to this knowledge gap, we conducted this study to extend the description of pathogens involved in fever with jaundice in the DRC, as well as to clarify the circulation of possible leptospirosis in the country. This study aims to evaluate the proportion and epidemiological characteristics of possible leptospirosis among patients with fever and jaundice in the DRC.

## Methods

### Ethics statement

This work was carried out as part of yellow fever surveillance organized by the DRC Ministry of Public Health in the Democratic Republic of the Congo through the Institut National de Recherche Biomedicale (INRB). This study was conducted with the permission of the DRC Ministry of Health to complement other studies conducted in the DRC to complete yellow fever surveillance data. The anonymity of the patients included in the yellow fever surveillance was respected; no written or verbal consent was obtained. However, this study was approved by the Institutional Review Board of the Nagasaki University Graduate School of Tropical Medicine and Global Health.

### Study design and participants

Since 2003, the DRC Ministry of Health, in collaboration with WHO, has set up a yellow fever surveillance system. In this system, case notification is based on a standard case definition and reporting by health districts. Suspected yellow fever cases are defined as any person with acute fever with jaundice occurring within 14 days of the onset of symptoms and not responding to antimalarial drugs or who tests negative for malaria by thick blood smear[19]. Blood samples from suspected yellow fever cases are collected and sent from health districts to the Institut National de Recherche Biomedicale (INRB) (Kinshasa, DRC), the country reference laboratory for yellow fever serological analysis.

Between 2017 and 2018, 1,562 suspected yellow fever cases were reported to INRB. Of these 1,562 cases, 70 were confirmed as yellow fever cases by serological analyses and 1,492 cases had negative serological results.

We retrospectively analyzed data and serum samples notified to the INRB from January 2017 to December 2018 for IgM presence against *Leptospira* spp. We used samples and data of suspected cases of yellow fever that had negative yellow fever serology, and which had available data and sufficient sample volume for biological analysis. A total of 1,300 subjects with available socio-demographic data and sufficient sample volume were included in this study.

The data was collected and reported from all the country’s health districts as part of the yellow fever surveillance in the DRC. All Data were stored on an Excel dataset (Microsoft Corporation, 2018. Microsoft Excel, Available at https://office.microsoft.com/excel.) and contains socio-demographic information of patients. The dataset did not have any information on clinical course evolution. In this database, all suspected cases and their biological specimens were assigned matching code numbers. The code linking each participant’s biological sample with the electronic data serves as the participant’s ID number. There was no personal identifying information on the database.

### Outcomes

The study’s outcome was an IgM anti-leptospiral antibody status that could either be positive (an individual with anti-*Leptospira* antibodies) or negative (an individual without anti-*Leptospira* antibodies). According to the WHO, a probable leptospirosis case is defined as a clinically compatible case with laboratory findings such as a positive result of an IgM ELISA or other rapid screening test[20].

### Procedures

Serology screening for IgM antibodies against *Leptospira* spp. was performed at the Institut National de Recherche Biomedicale in Kinshasa (INRB). We used as screening methods an ELISA against type IgM anti-*Leptospira* antibodies described by Kitashoji et al. and following WHO guidelines [21, 22]. Previous studies have suggested using this method for determining anti-*Leptospira* serum antibodies and for seroprevalence studies[23–25]. A set of serum of a hundred healthy controls was used to validate the use of this protocol in DRC. The mean+3 standard deviation optical density value of healthy controls was used as the cut-off limit (0.691).

In brief, ELISA plates were coated with the *Leptospira* biflexa serovar Patoc antigens according to WHO guidelines [22]. Plates were washed seven times with 200 μl per well of distilled water and then blocked with 200 μl per well of 20 mg/ml of BSA (bovine serum albumin) in TBST (Tris-buffered saline containing Tween 20) for 1.5 h at room temperature (RT). The plates were then washed three times with 300 μl TBST per well.

Patient serum samples were diluted 400-fold with ELISA buffer (TBST containing 10 mg/ml of BSA). After dilution, a total of 50 μl were distributed per well, and the plates were incubated for 1.5 h at RT. The plates were then rinsed four times with 200 μl TBST per well, then replaced with 50 μl per well of peroxidase-conjugated goat anti-human IgM solution (QED Bioscience) diluted 5000-fold with ELISA buffer and then incubated for one hour at RT. The goat anti-human IgM solution was then rinsed out as above. Subsequently, 50 μl of o-phenylenediamine dihydrochloride solution per well was added, and the plate was incubated for 2 minutes at room temperature. The reaction was stopped by adding 50 μl per well of 1 M sulfuric acid solution.

For each patient included in the study, the following data were collected: age, sex, date of onset of symptoms, and residence demographics.

### Statistical analysis

Relative frequencies and percentages were calculated for qualitative variables, while measures of central tendency and dispersion were determined for quantitative variables. An exploratory/descriptive approach was taken in investigating the association between potential risk factors (sex, age group, year of symptoms onset, living area and type of climate), for leptospiral infection. Thus, an initial analysis produced estimates of univariate odds ratios (ORs) for the potential risk factors using logistic regression, followed by multivariable logistic regression including all risk factors. Test results were considered statistically significant when the probability value was equal to or less than 0.05.

As prior knowledge about other tropical diseases suggested that different risk factors may be relevant dependent on living area, logistic regression analyses were also conducted separately for urban and rural residence. Data analysis was performed using Stata Corp (2017) Stata Statistical Software: Release 15. College Station, TX: StataCorp LLC. QGIS software (QGIS 3.14) was used to produce a map of the spatial distribution of positive leptospirosis IgM cases. We used Microsoft Excel (version 2016) to generate a seasonality histogram of putative leptospirosis cases.

## Results

Of the 1,592 samples collected in 25 of the 26 DRC provinces from January 2017 to December 2018 and notified in the national yellow fever surveillance database, a total of 1,300 samples that had a volume sufficient for biological testing and data were included in this retrospective study. The socio-demographic characteristics and risk factors are summarized in Table 1.

**Table 1:** Socio-demographic, climate characteristics, and risk factors associated with positive leptospiral serology in DR. Congo (2017–2018).

	Total	*Leptospira* IgM positive	*Leptospira IgM negative*	Crude OR	p-value	Adjusted OR	p-value
	**(N, %)**	**(n, %)**	**(n, %)**				
***All study population***	1300	88(6.8)	1212(93.2)				
***Sex***					< 0.001		< 0.001
Male	757 (58.2)	70 (79.6)	687 (56.7)	2.97 (1.74–5.06)		2.70 (1.57–4.65)	
Female	543 (41.8)	18 (20.4)	525 (43.3)	1		1	
***Age group***					< 0.001		< 0.001
0–9	515 (39.6)	20 (22.7)	495 (40.8)	1		1	
10–19	235 (18)	13 (14.7)	222 (18. 3)	1.44 (0.70–2.96)		1.24 (0.60–2.59)	
20–29	223 (17.2)	30 (34.1)	193 (15.9)	3.84 (2.13–7)		2.85 (1.54–5.30)	
30–39	132 (10.2)	8 (9.1)	124 (10.2)	1.59 (0.68–3.71)		1.32 (0.55–3.14)	
40–49	91 (7)	11 (12.5)	80 (6.6)	3.40 (1.57–7.37)		2.86 (1.28–6.35)	
>50	104 (8)	6 (6.8)	98 (8.1)	1.51 (0.59–3.97)		1.21(0.46–3.18)	
***Living area***					< 0.001		0.03
Rural	886 (68.2)	36 (40.9)	850 (70.2)	1		1	
Urban	414 (31.8)	52 (59.1)	362 (29.8)	3.39 (2.18–5.28)		1.91 (1.04–3.48)	
***Seasonal distribution of case***					0.171		0.03
Dry season	296 (22.8)	15 (17.1)	281 (23.2)	1		1	
Rainy season	1004 (77.3)	73 (82.9)	931 (76.8)	1.46 (0.83–2.60)		1 .94 (1.03–3.65)	
***Type of climate***					< 0.001		0.08
Humid subtropical	223 (17.2)	9 (10.2)	214 (17.7)	1		1	
Tropical rainforest	491 (37.7)	17 (19.3)	474 (39.1)	0.85 (0.37–1.94)		0.64 (0.25–1.58)	
Tropical savanna	586 (45.1)	62 (70.5)	524 (43.2)	2.81 (1.37–5.76)		1.46 (0.63–3.39)	

The overall prevalence of *Leptospira* IgM in this study was 7% (88 patients). 70 patients were excluded from the study because they tested positive for yellow fever, and 192 patients were excluded either because of lack of data or because they had insufficient sample volume to perform the laboratory analyses. People with anti-*Leptospira* antibodies ranged in age from 4 months to 86 years old, with a median age of 16 years. Thirty leptospiral IgM positive cases (34%) were found among the 20–29 years age group, followed by the 0–9 years age groups with 20 (23%) of positive cases.

There was a statistically significant association between age group and the presence of leptospiral IgM (p < 0.001). In particular, the odds of leptospiral IgM were significantly higher in the 20–29 years age group compared with the 0–9 years age group (Crude OR 3.84; 95% CI 2.13–7). Out of 88 positive leptospiral IgM cases, 18 (20%) were female and 70 (80%) were male, with a crude odds ratio of 2.97 in favor of males (95% CI 1.74–5.06). Fifty-two of the positive leptospiral IgM cases (59%) lived in an urban setting; and the odds of leptospiral IgM cases were higher in those living in urban compared to rural areas (Crude OR 3.39; 95% CI 2.18–5.28). Of the 88 positive leptospiral IgM cases, 50 cases (57%) lived in Kinshasa province.

Multivariable analysis using logistic regression (LR) analysis was performed including all socio-demographics characteristics. Male sex (Adjusted OR 2.70; 95% CI 1.57–4.65), 20–29 years age group (Adjusted OR 2.85; 95% CI 1.54–5.30) and living in an urban area, albeit less strongly, (Adjusted OR 1.91; 95% CI 1.04–3.48) remained statistically significant following adjustment for other factors and the association with the seasonal distribution of case group became stronger in effect size as well as statistically significant (Adjusted OR 1.94; 95% CI 1.03–3.65).

A sub-analysis of data regarding living areas showed greater odds of displaying positive leptospiral IgM for males than females in the urban (Crude OR 4.60; 95% CI 2.01–10.74) compared to rural areas (Crude OR 1.78; 95% CI 0.86–3.6)); However, this association was not statistically significant. The highest positivity rate of *Leptospira* IgM was found in the 20–29 years age group (42%) in the urban areas, while the highest positivity rate in the rural areas was found in the 0–9 years age group (36%). In both urban and rural areas, most of the cases occurred during the rainy season. However, the odds of finding positive leptospiral IgM were in favor of the rainy season rather than the dry season only in the urban setting (Crude OR 2.96; 95% CI 1.22–7.716). Besides the rainy season (Adjusted OR 3.34; 95% CI 1.34–8.29), multivariable logistic regression showed that male sex (Adjusted OR 4.41; 95% CI 1.87–10.39) and rainy season (Adjusted OR 3.14; 95% CI 0.38–25.49) were associated with positive leptospiral IgM in the urban setting. However, we note in general that the measures of effect that we estimated are fairly imprecise (with very large confidence intervals) in the subgroup analyses by living area due to the reduced sample size. The socio-demographic characteristics and risk factors of this sub-analysis are summarized in Tables 2 and 3.

**Table 2:** Socio-demographic, climate characteristics, and risk factors associated with positive leptospiral serology in urban areas of the DR. Congo (2017–2018).

	Total	*Leptospira* IgM positive	*Leptospira* IgM negative	Crude OR	p-value	Adjusted OR	p-value
	**(N, %)**	**(n, %)**	**(n, %)**				
***All study population***	414	52 (12.6)	362 (87.4)				
***Sex***					< 0.001		< 0.001
Male	256 (61.8)	45 (86.5)	211 (58.3)	4.60 (2.01–10.47)		4.41 (1.87–10.39)	
Female	158 (38.20	7 (13.4)	151 (41.7)	1		1	
***Age group***					< 0.001		0.02
0–9	119 (28.7)	7 (13.4)	112 (30.9)	1		1	
10–19	87 (21)	8 (15.4)	79 (21.8)	1.62 (0.56–4.65)		1.73 (0.58–5.12)	
20–29	95 (23)	22 (42.3)	73 (20.2)	4.82 (1.96–11.86)		3.55 (1.40–8.98)	
30–39	45 (10.9)	7 (13.5)	38 (10.5)	2.94 (0.97–8.94)		2.29 (0.72–7.20)	
40–49	29 (7)	6 (11.5)	23 (6.4)	4.17 (1.28–13.57)		3.52 (1.03–12)	
>50	39 (9.42)	2 (3.9)	37 (10.2)	0.86 (0.17–4.34)		0.65 (0.12–3.37)	
***Seasonal occurrence of case***					< 0.001		< 0.001
Dry season	107 (25.9)	6 (11.5)	101 (27.9)	1		1	
Rainy season	307 (74.1)	46 (88.5)	261 (72.1)	2.96 (1.22–7.16)		3.34 (1.34–8.29)	
***Type of climate***					0.11		0.2
Humid subtropical	25 (6)	1 (1.9)	24 (6.6)	1		1	
Tropical rainforest	11 (2.7)	0 (0)	11 (3)	0 (0)		0(0)	
Tropical savanna	378 (91.3)	51 (98.1)	327 (90.4)	3.74 (0.49–28.27)		3.14 (0.38–25.49)	

**Table 3:** Socio-demographic, climate characteristics, and risk factors associated with positive leptospiral serology in rural areas of the DR. Congo (2017–2018).

	Total	*Leptospira* IgM positive	*Leptospira* IgM negative	Crude OR	p-value	Adjusted OR	p-value
	(N, %)	(n, %)	(n, %)				
***All study population***	886	36 (4.1)	850 (95.9)				
***Sex***					0.104		0.109
Male	501 (56.5)	25 (69.4)	476 (56)	1.78 (0.86–3.6)		1.77 (0.85–3.68)	
Female	385 (43.5)	11 (30.6)	374 (44)	1		1	
***Age group***					0.188		0.181
0–9	396 (44.7)	13 (36.1)	383 (45.1)	1		1	
10–19	148 (16.7)	5 (13.9)	143 (16.8)	1.03 (0.36–2.94)		1.(0.36–2.99)	
20–29	128 (14.5)	8 (22.2)	120 (14.1)	1.96 (0.79–4.85)		1.97 (0.78–4.92)	
30–39	87 (9.8)	1 (2.8)	86 (10.1)	0 .34 (0.04–2.65)		0 .35 (0.045–2.74)	
40–49	62 (7)	5 (13.9)	57 (6.7)	2.58 (0.88–7.52)		2.65 (0.90–7.81)	
>50	65 (7.3)	4 (11.1)	61 (7.2)	1.93 (0.61–6.11)		2.07 (0.64–6.61)	
***Seasonal occurrence of case***						
Dry season	189 (21.3)	9 (25)	180 (21.2)	1	0.590	1	0.736
Rainy season	697 (78.7)	27 (75)	670 (78.8)	0.80 (0.37–1.74)		0.83 (0.29–2.36)	
***Type of climate***					0.581		0.736
Humid subtropical	198 (22.3)	8 (22)	190 (22.4)	1		1	
Tropical rainforest	480 (54.2)	17 (47.2)	463 (54.4)	0.87 (0.37–2.05)		0.98 (0.31–3.09)	
Tropical savanna	208 (23.5)	11 (30.6)	197 (23.2)	1.32 (0.52–3.36)		1.57 (0.55–4.51)	

Of the 26 provinces within the country, 25 were represented in the national yellow fever surveillance dataset for the study period, and anti-*Leptospira* antibodies were found in samples collected from 14 provinces. The distribution of DR. Congo provinces by leptospiral status are shown in Table 4. The province with the highest percentage of positive samples for *Leptospira* IgM was Nord-Kivu (14.7%), followed by Mongala (14.3%) and Kinshasa (14.2%) provinces. 50 out of 88 positive leptospiral IgM cases were in the province of Kinshasa.

**Table 4:** Distribution of DR. Congo provinces by leptospiral serology status (2017–2018).

Province of residence	*Leptospira* IgM positive	*Leptospira* IgM negative	Total
	(n, %)	(n, %)	(N, %)
All study population	88 (6.8)	1212 (93.2)	1300 (100)
Bas–Uele	3 (3.2)	92 (96.8)	95 (100)
Equateur	1 (1.9)	53 (98.1)	54 (100)
Haut–Lomami	7 (8.1)	79 (91.9)	86 (100)
Haut—Uele	0 (0)	11(100)	11 (100)
Haut- Katanga	0 (0)	7 (100)	7 (100)
Ituri	0 (0)	8 (100)	8 (100)
Kasai	0 (0)	20 (100)	20 (100)
Kasai Central	0 (0)	30 (100)	30 (100)
Kasai Oriental	0 (0)	1 (100)	1 (100)
Kinshasa	50 (14.2)	301 (85.8)	351 (100)
Kongo Central	1 (2)	49 (98)	50 (100)
Kwango	4 (5.2)	73 (94.8)	77 (100)
Kwilu	1 (2.4)	40 (97.6)	41(100)
Lomami	0 (0)	22 (100)	22(100)
Lualaba	0 (0)	60 (100)	60 (100)
Maindombe	0 (0)	4 (100)	4 (100)
Maniema	0 (0)	1 (100)	1 (100)
Mongala	1 (14.3)	6 (85.7)	7 (100)
Nord—Kivu	5 (14.7)	29 (85.3)	34 (100)
Nord—Ubangi	6 (11.1)	48 (88.9)	54 (100)
Sud—Kivu	1 (12.5)	7 (87.5)	8 (100)
Sud—Ubangi	3 (13)	20 (87)	23 (100)
Tanganyika	2 (11.8)	15 (88.2)	17 (100)
Tshopo	0 (0)	5 (100)	5 (100)
Tshuapa	3 (1.3)	231 (98.7)	234 (100)

**Fig 1:**
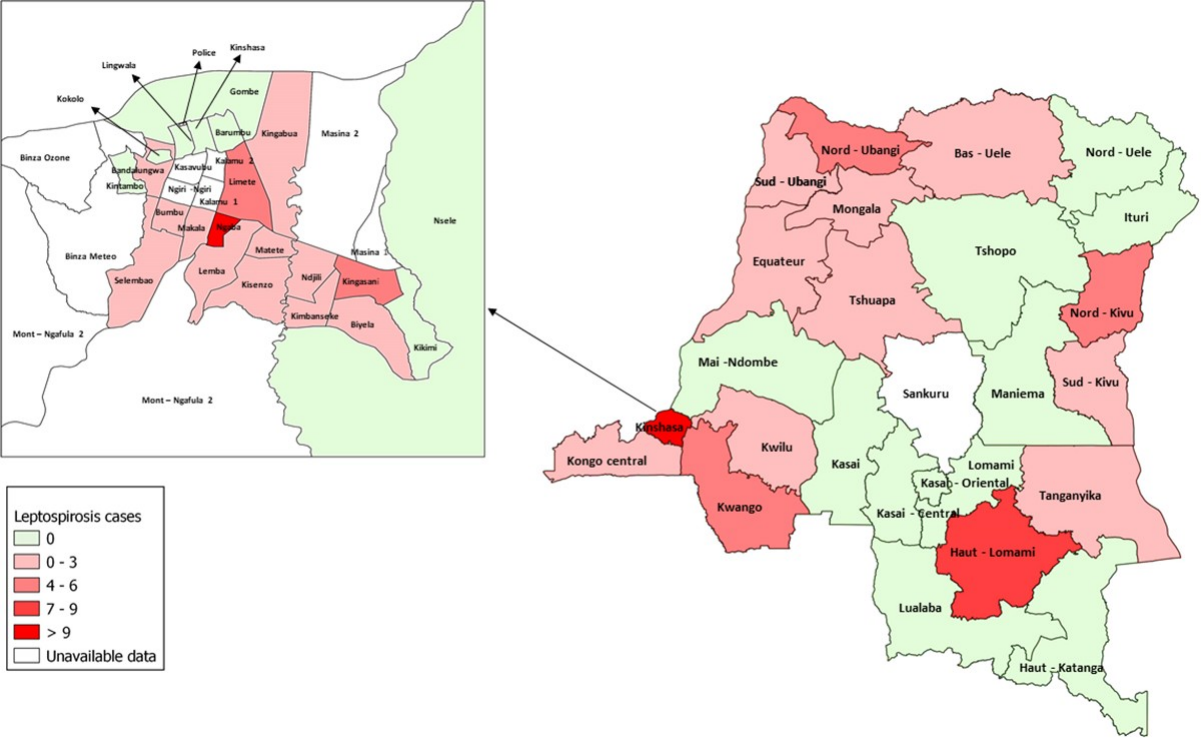
Map of positive leptospiral IgM cases distribution by province of the Democratic Republic of the Congo. The provinces in which positive cases were detected are shaded in red, and provinces are shaded in green that reported suspected cases of yellow fever. (Source: The map was created with the provincial Shapefile obtained from the free, open, collaborative platform Common geographical reference of DRC (https://datacatalog.worldbank.org/dataset/democratic-republic-congo-administrative-boundaries-2017) accessed on April 25th,2020. The map was created using QGIS software (QGIS 3.14) geographical information system).

The geographic distribution of the 88 IgM leptospiral cases per province is shown in Fig 1. Out of 88 positive leptospiral IgM cases, 62 (71%) were found in areas with a tropical savanna climate. The odds of finding *Leptospira* IgM was almost three times higher in areas with tropical savanna climate compared to areas with humid subtropical climate (Crude OR 2.81; 95% CI 1.37–5.76).

**Fig 2:**
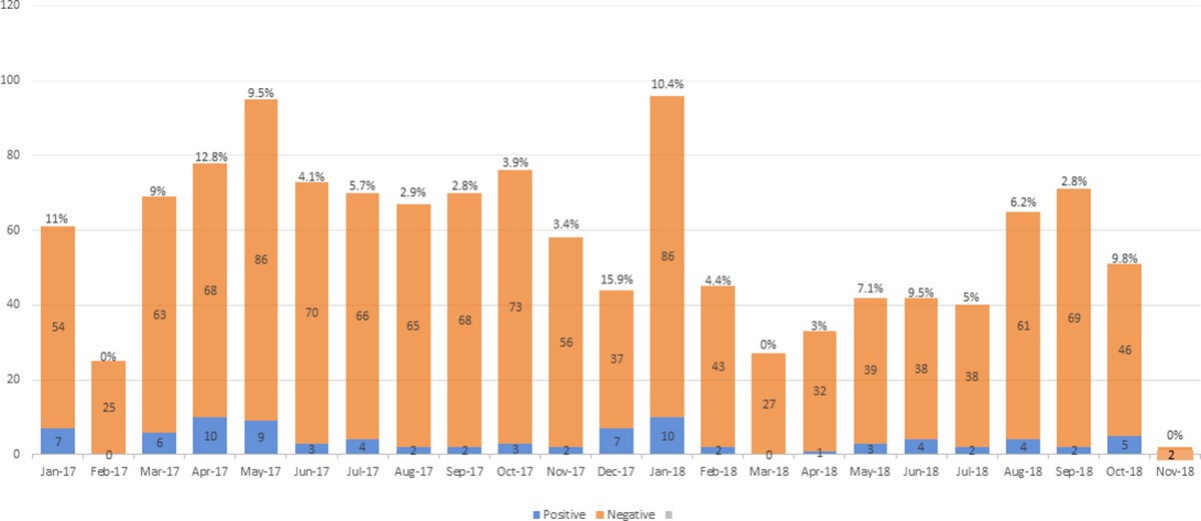
Seasonal trend of leptospiral IgM positivity rate within the DR. Congo national network for yellow fever surveillance (2017–2018). Blue and Orange charts indicate the number of positive leptospiral IgM cases and the number of specimens tested. The percentage on top of each chart indicate the positivity rate for leptospiral IgM of the month.

The seasonal distribution of leptospiral IgM positive indicated greater odds of positive cases among samples received during the rainy season compared with the dry season, although these were not statistically significant (Crude OR 1.46; 95% CI 0.83–2.37). Seventy-nine percent of the IgM positive cases occurred during the rainy season. The seasonal distribution of the 88 leptospiral IgM positive cases by month for the years 2017 and 2018 is shown in Fig 2.

## Discussion

In this retrospective analysis of samples from yellow fever surveillance in the DRC, we found that 7% of suspected cases with negative serology had IgM against *Leptospira spp*. The majority of these *Leptospira* IgM cases were among young adult males (20–29 years) living in an urban setting. The highest percentage of positive were found in the province of Nord Kivu province (14.7%) followed by the province of Mongala (14.3%) and the province of Kinshasa (14.2%) These data suggest that *Leptospira* spp. are a probable cause of fever and jaundice in the DRC.

Studies conducted on *Leptospira* spp. as a cause of fever with jaundice in other African countries reported an overall seroprevalence of 8% in the Central Africa Republic, 6% in Burkina Faso, 8% in Ghana, and 9% in Ivory Coast. Our data are in line with these seroprevalences as well as like publications in African countries that have reported that young adult males in the 20–29 years group (in the working-age) are at risk of leptospirosis because of their occupational exposures. Our findings further highlighted a high proportion of positive cases among the 0–9 years age group (23%). Zida et al. found a similar trend in Burkina Faso, and the fact that 60% of the Congolese population age is under 20 years old could explain this trend [10, 11, 15, 16, 26].

Regarding individuals with *Leptospira* IgM seropositivity, we found that 59% of positive cases lived in an urban setting, and 41% lived in a rural setting. This pattern is consistent with the literature that reports that leptospirosis occurs in both rural and urban settings in tropical and sub-tropical countries [27]. Moreover, our data concur with the literature that suggested that leptospirosis could spread from its traditional environment in rural areas to reach urban areas with poor sanitation infrastructure [28–31]. Leptospirosis is globally known as an urban health problem and the role of urban areas in the re-emergence of leptospirosis has been described by several authors [31–33]. The high seroprevalence in urban settings may relate to poor urbanization (increased risk of flooding), overcrowded populations, as well as knowledge, attitude, and practice determinants. A limitation of this study is that the limited number of cases did not allow us to provide precise measures of effect with respect to the risk factors. Further larger studies are needed to identify risk factors and the true burden of the disease in rural and urban settings in the DRC.

The Democratic Republic of Congo (DRC) is a large African country that covers approximately 2.3 million square kilometers. The vast territory of the DRC spans multiple climate and vegetation types. The climate is hot and humid with two different types of seasons (Rainy and Dry season) with different duration depending on the type of climate and geographical location[34]. The geographical distribution of leptospiral IgM seropositivity showed that 25 out of the 26 of the DRC provinces were included in this study and our study population was representative of the country. Subjects with anti-*Leptospira* IgM antibodies were found in 14 provinces with different type of climate, suggesting a broad distribution and circulation of *Leptospira spp*. in the country. The epidemiology of leptospirosis varies significantly from one geographical area to another, depending on the climate and lifestyles of the inhabitants. We reported that 50 out of 88 positive cases were in the city province of Kinshasa. Kinshasa is the most populous province in the DRC with an approximate population close to 14 million people[35]. Kinshasa is facing rapid urbanization with an overcrowding population and is prone to flooding after heavy rains. It is described that rapid urbanization, overcrowding, and natural disasters are risk factors promoting the transmission of leptospirosis [36, 37].While the provinces of North Kivu and Mongala showed the highest rates of positivity, their interpretation remains difficult because the analyses were based on a small number of samples compared to those from Kinshasa. Further studies are required to clarify the burden of leptospirosis in these provinces.

The seasonality of leptospirosis has been described in most parts of the world, with a significantly higher incidence during rainy months, in tropical areas [38, 39]. We found that cases occur year-round in the DRC, with a higher proportion during the rainy season. The climate of the DRC is diverse, with equatorial type, warm, humid in the center of the country, and tropical to the south and north. Rainfall is regular and abundant but varies in time and space. Further studies taking into account climatic and geographic variations as well as rainfall will help to better understand and explain the epidemiological link between the seasonal distribution of cases, climate and leptospirosis in DRC.

The classical risks factors such as male sex, living in an urban area, and age group were associated with positivity to IgM against *Leptospira* in multivariable analysis. These findings are consistent with studies identifying *Leptospira* risk factors[9, 27, 40]. The significant associations with male sex are consistent with the fact that most occupations at high risk (farmers, miners, recreational activities in muddy grounds, exposures to flood water, building workers) for leptospirosis are often associated with males.

Epidemiological surveillance of yellow fever in the DRC has been in effect since 2003. This study showed for the first time that 7% of suspected cases of yellow fever had leptospiral sero-positivity across the urban and rural health districts of the DRC. The availability of socio-demographic data enabled us to describe the epidemiology of the disease as well as its geographical distribution. More studies focusing on seroprevalence, risk factors, and geographical distribution of leptospirosis cases are needed in the DRC.

This study has several limitations. This study was a retrospective study based on the analysis of a single sample. Thus, this study may have underestimated leptospirosis cases because a second sample was not tested and more specific and sensitive laboratory tests such as identification of circulating serogroups by MAT (Microscopic Agglutination Test) and PCR were not performed. Several authors have recently recommended combining serological tests with PCR (Polymerase Chain Reaction). PCR is a complementary test, especially when no specific antibodies were detected by serological methods and allows to detect more cases than a serological test would have missed [41–43]. We believe that this also contributed to the underestimation of the results reported in this study.

The lack of information on the clinical course and other symptoms of included patients and the failure to systematically capture all suspected cases are limitations of the study. Despite these limitations, this study shows that *Leptospira* spp. are one of the possible causes of fever with jaundice in the DRC. It complements other studies conducted to identify other infectious causes of fever with jaundice in the DRC. Surveillance should be expanded to look for other infectious causes of febrile jaundice. There is a need to expand the panel of laboratory diagnostic pathogens screened within the surveillance network.

## Conclusion

This retrospective study using samples collected for yellow fever surveillance, found that amongst those with negative yellow fever serology, 7% had anti-*Leptospira* IgM. This finding suggest that leptospirosis is a possible cause of fever with jaundice in the Democratic Republic of the Congo, and it should be considered in the differential diagnosis of fever with jaundice. Testing for leptospirosis should be included in the panel of pathogens screen when investigating a case of fever with jaundice. Further studies are needed to identify animal reservoirs, associated risk factors, and the actual burden of human leptospirosis in the DRC. Awareness of leptospirosis among clinicians, funding for new studies, and the possibility of validating laboratory diagnostic tests in the field are needed to clarify the extent of the problem in the DRC.
